# Care challenges related to lack of awareness in people with dementia

**DOI:** 10.3389/frdem.2026.1770977

**Published:** 2026-02-12

**Authors:** Catherine M. Alexander, Linda Clare

**Affiliations:** 1 REACH: The Centre for Research in Ageing and Cognitive Health, University of Exeter Medical School, Exeter, United Kingdom; 2 NIHR Applied Research Collaboration South West Peninsula, Exeter, United Kingdom; 3 NIHR Policy Research Unit in Dementia and Neurodegeneration Exeter (DeNPRU Exeter), Exeter, United Kingdom

**Keywords:** Alzheimer’s, anosognosia, awareness, caregiver, consultation, homecare, person-centered, stakeholder

## Abstract

**Introduction:**

Person-centered care can be difficult to provide where people with dementia lack awareness of dementia-related changes. They may appear reluctant to discuss changes or unable to recognize care needs. Lack of awareness can contribute to worse outcomes for people with dementia and caregivers. However, little is known about how to manage care challenges arising from lack of awareness.

**Methods:**

Stakeholder perspectives were sought about experiences around lack of awareness in people with dementia living at home. Discussion groups informed content of an online survey for informal caregivers, clinicians and homecare professionals. Additional information came from secondary analysis of interviews with people with dementia. Data were analyzed descriptively, with content analysis used to categorize qualitative information.

**Results:**

The survey was completed by 54 people involved in dementia care. Most had encountered lack of awareness, with 75% encountering it often or extremely often. Lack of awareness led to delays in referrals, diagnosis and starting care packages, with disagreements commonly experienced, both at home and in clinical settings. There were concerns about safety and safeguarding, and in some cases complete breakdown of care at home. Management involved a flexible and patient approach, avoiding confrontation where possible. Interview transcripts from people with dementia demonstrated how awareness affects strategies used and thinking about the future.

**Discussion:**

The consultation highlighted the importance of this topic and implications for care provision. More information for the general public and educational resources for informal caregivers and professionals would improve understanding of awareness issues and sharing of effective strategies.

## 1 Introduction

Best practice in dementia care involves a person-centered approach, considering the individual’s needs and preferences (National Institute for Health and Care Excellence (NICE), 2018). These can be difficult to determine if the person with dementia denies any problems or seems unaware of any decline in abilities. This can make it challenging to openly talk about their needs and the kind of help that might be useful. However, little is known about how to manage situations related to lack of awareness.

Awareness can be defined as a reasonable or realistic perception or appraisal of a given aspect of one’s situation, functioning or performance, or of the resulting implications, expressed explicitly or implicitly (Clare et al., 2011a). This broad definition can be used in reference to reduced awareness due to neurological disease, often referred to as anosognosia, and that mediated by psychological processes including denial and/or avoidant coping styles. It can be difficult to distinguish between the causes (Prigatano et al., 2024), but both result in situations relevant to dementia care. Estimates of the prevalence of awareness problems, range widely from 21 to 81% in mild dementia (Sunderaraman and Cosentino, 2017). Proposed models attempt to explain the processes involved in awareness (Morris and Mograbi, 2013), the impact of pathology (Hanseeuw et al., 2020; Mograbi et al., 2021), or the influence of psychosocial factors (Clare et al., 2011a). Disease progression has been associated with worsening awareness (Turró-Garriga et al., 2016), and lack of awareness in early stages of cognitive impairment may predict prognosis in Alzheimer’s disease (López-Martos et al., 2024).

Lack of awareness can have an impact on caregivers, with increased burden and costs of dementia care (Turró-Garriga et al., 2013; Perales et al., 2016; Turró-Garriga et al., 2016). There can also be worse outcomes for people with dementia regarding risk of dangerous behavior and more rapid institutionalization (Starkstein et al., 2007; Parrao et al., 2017). However, data on outcomes remain limited due to paucity of clinical assessments of awareness. The lack of suitable clinical tools is unhelpful (Alexander et al., 2021), although new tools are in development (Pennington et al., 2025).

Better awareness can enable individuals to make use of cognitive rehabilitation programs (Fernández-Calvo et al., 2015; Dutzi et al., 2019). However, there is no indication that forcing acknowledgement of dementia symptoms or diagnosis would be helpful for an individual. Quality of life, wellbeing and mood may be worse for people with greater awareness of their difficulties (Azocar et al., 2021; Stites et al., 2017). This may be particularly distressing for people with young-onset dementia (Baptista et al., 2021), for whom dementia-related difficulties can have greater impact on maintaining activities such as driving, employment and family responsibilities.

Although lack of awareness is frequently observed in people with dementia, there is a lack of published research about strategies and interventions to manage difficult situations that arise (Alexander et al., 2025). This paper reports findings from a stakeholder consultation project designed to identify the implications of lack of awareness for everyday dementia care provision. The over-riding research question was: How can we improve person-centered care for people living with dementia who appear unaware of their dementia-related difficulties? Through small group discussions and a wider online survey, informal caregivers, clinicians, and homecare/social care professionals were asked about their experiences in caring for people with dementia who live at home. The aim was to find out the extent of the problem, the nature of the problems encountered, and best ways of managing situations arising from lack of awareness. This would help to identify areas where more supportive interventions are needed.

The findings have been supplemented by information from previous interviews with people with mild-to-moderate dementia conducted in an earlier study (Clare et al., 2011b) to provide further context and, where available, illustrate their views about awareness of dementia. A patient and public involvement and engagement (PPIE) group provided insights into the topic area and reviewed the proposed survey questions.

## 2 Methods

Study design: An exploratory design was employed to gather and report views from stakeholders involved in dementia care. It comprised three stakeholder consultation groups, an online survey, and secondary analysis of an existing dataset of interviews with people with dementia. The consultation groups were designed to inform the content of the survey, but not to generate qualitative data. Analysis of the results is based on the survey data and secondary analysis of the existing dataset. The work was conducted from a UK research center, with involvement of stakeholders throughout the UK.

### 2.1 Small group consultations

Separate group meetings were held in person with informal caregivers providing unpaid care at home (also referred to as ‘carers’), and online with clinicians, and with homecare/social care professionals providing paid care. These people were invited from stakeholder organizations known to the research team, i.e., caregiver support networks, homecare professional forums and dementia clinical research networks. Two of these people were already familiar with the awareness research. The informal caregivers’ group comprised five people with current or past experience as a dementia caregiver. The clinicians’ meeting involved a consultant geriatrician and three people from a nursing background who work in senior positions within dementia post-diagnostic support services. Meetings for homecare/social care professionals were held with two senior members of staff from homecare organizations and a social care researcher with extensive experience in care provision. Each discussion was guided by semi-structured questions on a range of issues related to lack of awareness relevant to that area of dementia care, in consideration of draft questions for the survey. Handwritten notes were taken by the researcher during the meetings and cross-checked with an audio-recording of the meeting to ensure that all issues raised had been documented. This information was not formally analyzed but was used to guide the formation of the survey questions, which were then finalized in consultation with the PPIE group.

### 2.2 Online survey

The anonymous survey was distributed digitally through known contacts in networks for clinical research, homecare research, and caregiver support. A flyer containing the survey link was shared through the usual communication channels for advertising research opportunities, which included online bulletins, digital newsletters, and at in-person meetings. Survey responses were only collected if all consent statements had been checked at the start of the survey, and decision to submit responses was checked at the end of the survey. The survey was live for 8 weeks from June 23rd to August 18th, 2025, using the Qualtrics platform. Questions were tailored to either informal caregivers, clinicians, or homecare/social care professionals. Initial questions about demographic information were followed by seven Likert-type questions for each group and three free text questions about awareness; see Supplementary material. For the informal caregivers, additional demographic questions were included about the cared-for person.

The demographic information was used to describe the survey respondents. For the Likert-type questions about awareness, results are presented descriptively with frequency of responses shown in bar charts created for each group. Where questions were comparable, responses for the groups are shown in combined charts. Free text responses were examined for each question separately and for each group of respondents, using an Excel spreadsheet. Each respondent was assigned a number so that the original data could be identified and checked. Working iteratively, responses were summarized and sorted into categories representing an area of concern reported by the respondents. Individual responses were tabulated, with rows showing the area of concern, and columns showing relevant responses to each question, i.e., main concerns and worst consequence, strategies and suggestions for managing problems, and what would help. By organizing the data in categories, areas of concern could be identified and described. This descriptive content analytical approach comprehensively demonstrated the concerns and strategies reported by the respondents. Individual quotes from the free text responses have been used to illustrate the findings.

Ethics approval was provided by the University of Exeter Medical School Research Ethics Committee, reference number 8487474.

### 2.3 Previous interviews with people with dementia

We wanted to represent people with dementia in the consultation, but it was considered inappropriate to gain their views through an anonymous online survey, with no prior knowledge of their perception of their condition. Instead, a secondary analysis of an existing dataset (Clare et al., 2011b) was used. Interviews with 101 people with mild-to-moderate dementia were conducted as part of the Memory Impairment and Dementia Awareness Study (MIDAS; Clare et al., 2011b). This available dataset collected in 2008 was extensive and suitable for purpose, and it continues to be relevant for the phenomenon under discussion. MIDAS received ethical approval from Wales Research Ethics Committee 5 (reference 05/WNo01/45). Participants were recruited through memory clinics in North Wales, and informed consent was given by all participants. Approval for unrestricted secondary use of the anonymized data by researchers new to the research team was confirmed by Wales Research Ethics Committee 5 in 2016.

Memory Impairment and Dementia Awareness Study data included, among other awareness measures, a global awareness rating for each participant based on qualitative analysis of interviews with the person with dementia and a caregiver. Awareness was classified with a five-point rating scale ranging from 1 (no evidence of awareness) to 5 (extensive awareness). Here, interview transcripts have been studied for the four people judged as showing no evidence of awareness, in comparison with four cases matched by age and gender, who were judged to show extensive awareness. Having identified the matched cases, the content of the interview transcripts was examined by the first author. Using a spreadsheet, responses for each participant were tabulated in rows, initially using broad areas from the interview questions as column headings, i.e., on visiting the memory clinic, reflection on diagnosis, noticing changes in memory, on changes in abilities, strategies, on need for support, disagreements, anything worrying you, about the future. The information from participants in each group was compared and summarized, with quotes used to illustrate the differences regarding apparent awareness of the diagnosis and its implications, use of strategies and thinking about the future. This focused approach was used to provide perspectives and show differences between people with dementia with greater or lesser awareness of their difficulties, using their own words. Transcripts for a further 10 people with extensive awareness were examined for any reflections about awareness, i.e., reflections expressed by the person with dementia about being aware of symptoms or the diagnosis.

## 3 Results

**Figure 1:**
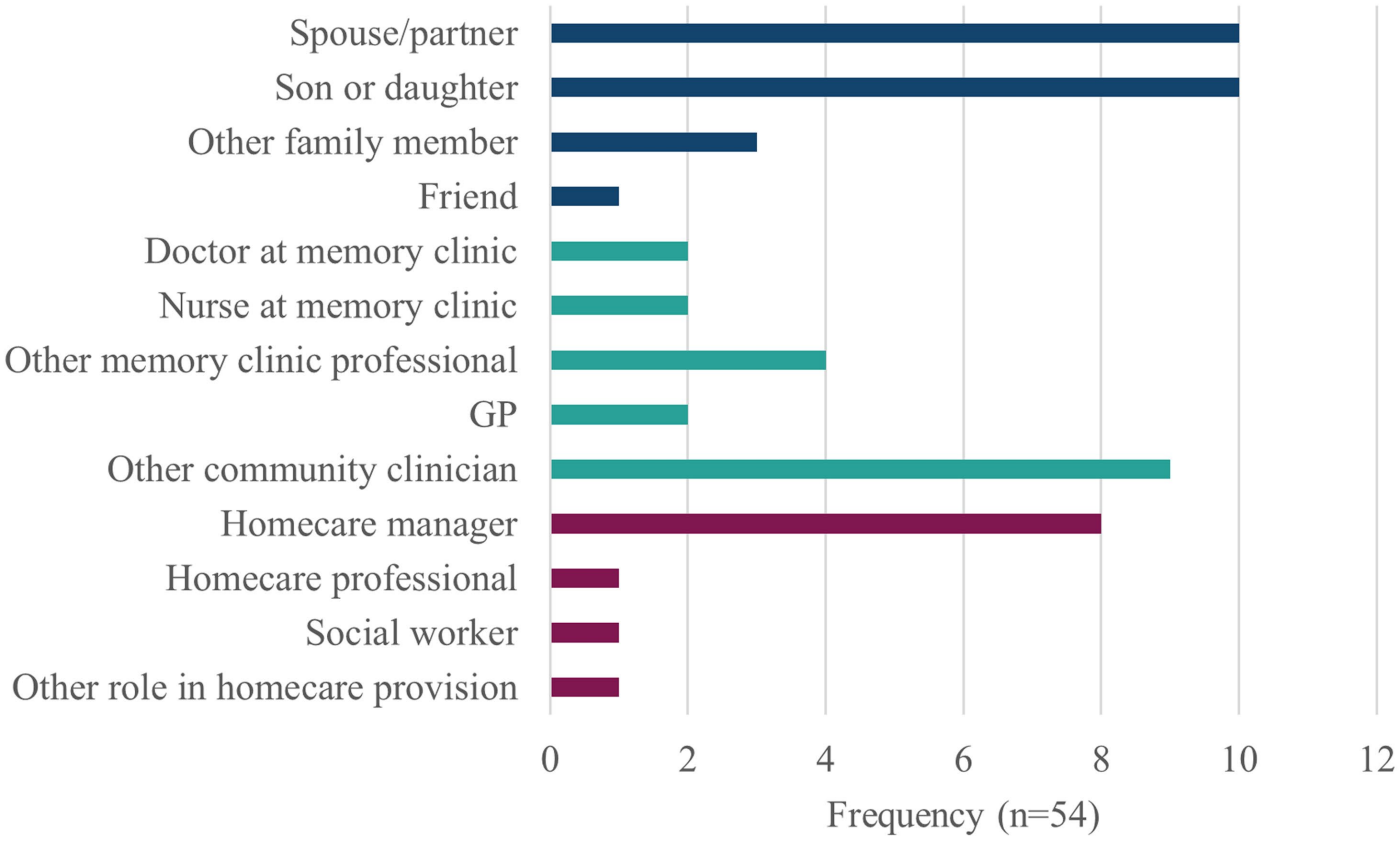
Role of survey respondents. Other roles: other memory clinic professionals were clinical psychologists (*n* = 3) and a dementia specialist. Other community clinicians were clinical psychologists (*n* = 2), community nurses working in dementia care or mental health (*n* = 5), a social prescriber (*n* = 1), and an occupational therapist. Homecare professionals included one dementia support worker.

The survey was completed by 54 respondents, i.e., 24 informal caregivers, 19 clinicians and 11 homecare/social care professionals (referred to as ‘homecare’), with a range of roles in care provision; see Figure 1.

The majority of respondents were female, from white ethnic groups representing nine UK regions. The informal caregivers were generally older than the clinicians or homecare professionals; 11/24 caregivers lived with the person they cared for, of whom 10 were spouses. The informal caregivers cared for 13 males and 11 females with dementia who were mostly in age groups 70–79 or 80 and above, all in ethnic group white.

Two clinicians worked only in care homes. Their responses are reported separately as the project focused on care for people with dementia who live at home; see the full report (Alexander, 2025).

### 3.1 Lack of awareness encountered in dementia care

**Figure 2:**
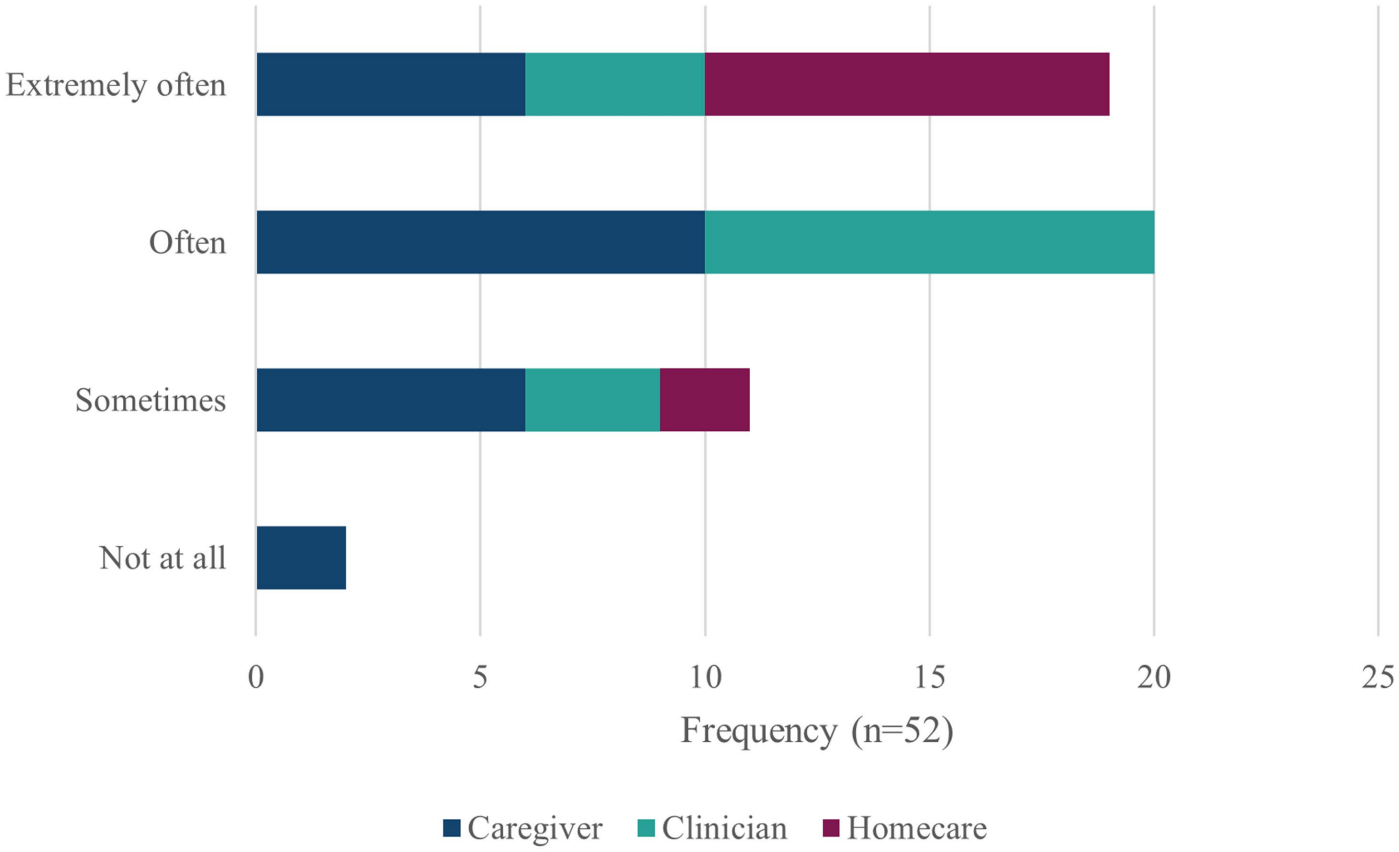
Encountering lack of awareness.

The initial consultation meetings indicated that lack of awareness was frequently encountered and often problematic, and this was confirmed in the survey responses; see Figure 2. Nearly all the people responding had encountered lack of awareness; 75% encountering it often or extremely often.

Three informal caregivers were unsure whether the person they cared for was currently aware of the diagnosis of dementia, 10/24 reported them as not aware, and 11/24 reported them as being aware of the diagnosis. Two of these reported that the problem with lack of awareness related to the primary caregiver, not the person with dementia. Their responses to the remaining questions are reported separately (Alexander, 2025).

Even if the diagnosis was acknowledged, there was sometimes lack of awareness of changes in abilities due to dementia.

*“Is aware she has dementia but thinks she is invincible”.*

Clinicians agreed that lack of awareness was a common and significant problem in dementia care.

*“Anosognosia is a common feature in dementia and often affects multiple elements of the person’s diagnosis, treatment, care and overall wellbeing.”*

*“Significant effect on accessing appropriate help and support for the person and carers.”*

Homecare professionals observed that denial is frequently encountered, but the use of early coping strategies such as calendars and memory aids might indicate an underlying recognition of dementia-related changes in some individuals, even if not overtly acknowledged.

### 3.2 Main problems and consequences

#### 3.2.1 Delays

Delays in the care pathway related to lack of awareness were reported by most respondents. Five informal caregivers reported a delay in referral of more than a year, due to individuals’ refusal to attend the GP for an initial assessment or not attending follow-up. One caregiver felt that the lack of acceptance of the condition may have made the situation more “…*terrifying and isolating*” for that person, whose health suffered due to not seeking support earlier. Clinicians confirmed that late presentation to memory clinic is common; one clinician estimated a delay of 6–8 months on average between initial referral and the repeat referral.

*“Family want them to go (for assessment), and they think nothing is wrong”.*

When care is offered, the care package is commonly rejected if the person does not recognize or accept their difficulties, and/or believes they are managing adequately without support.

*“For example, patients believing that they go out and do all their own shopping when they have not left the home.”*

Homecare professionals describe how care is commonly initiated late, in response to a crisis, and the window for facilitating more independent living is missed. Individuals may completely refuse the service offered, deny access to their home, or refuse to pay. This can result in the family trying to manage complex needs with inadequate support.

#### 3.2.2 Disagreements and conflicts

Nearly all respondents (90%) reported an area of disagreement related to lack of awareness, in particular affecting acceptance of the diagnosis or around the type of homecare needed. Caregivers shared how this can result in the individual with dementia getting angry and not understanding the need for things to be done differently. Clinicians have observed significant challenges and friction for family members who are trying to offer help or address safety concerns. Irritability and verbal or physical aggression can arise due to perceived over-involvement and control over daily activities by a family member, for example when changing systems for payments and establishing a key safe for caregivers’ use. Similarly, homecare professionals have experienced conflict if the person becomes defensive about their needs and annoyed with the care-worker.

For clinicians, discussions about the diagnosis and medication can frequently be difficult; seven clinicians reported this happens often or very often. Sometimes, the diagnosis is met with disbelief and questioning of the doctor’s judgment, or outrage is directed toward the doctor. Post-diagnostic support and medication are rejected as believed to be unnecessary. There can be poor self-management of comorbid conditions such as diabetes, or increased risk of falls due to denial of mobility problems and a refusal to use aids. Planning for the future and discussing end of life care is often resisted.

Social behavior can be affected by lack of awareness, and informal caregivers frequently endorsed concerns over the person’s emotional response to others, for example, making disinhibited comments in public and not being aware that this has caused offense. One caregiver described the individual’s lack of understanding of the caregiver’s own health condition and needs.

#### 3.2.3 Safety or safeguarding concerns

**Figure 3:**
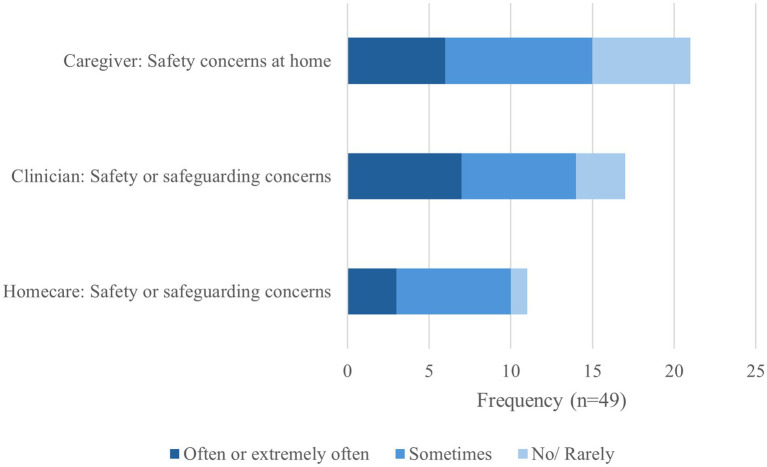
Safety or safeguarding concerns.

Concerns were endorsed by the majority of respondents; see Figure 3. Caregivers expressed many concerns, most commonly about safety of going out alone.

*“Thinks they are still able to do everything they used to, but I can see they aren’t always safe doing so.”*

Regarding driving, awareness difficulties not only affected safe handling of the vehicle but included awareness for navigation and making appropriate decisions before embarking on a journey. In the home, safety concerns were around cooking, using sharp objects or power tools for gardening or DIY, or concerns about being taken advantage of, particularly online. Clinicians also reported the dangers of online financial scams or fake relationships. At times, there can be very serious risks of financial, physical or sexual abuse. Homecare professionals described medication errors due to inaccurate self-administration, neglect of care needs regarding continence and neglect of skin care leading to skin breakdown. Consequences can be particularly dangerous for people with dementia who live alone.

In some cases, lack of awareness was thought to have contributed to the breakdown of homecare, acknowledged by the majority (21/27) of clinicians and homecare professionals. Caregivers admitted the strain that results from their role. The need for constant oversight and checking can be a source of frequent stress and becomes exhausting. In one case, a move to residential care resulted perhaps sooner than would have been needed if help had been accepted earlier by the person with dementia.

### 3.3 Strategies and suggestions

All respondent groups described using a person-centered approach carried out with kindness and understanding, acknowledging the individual’s identity as a person, not just as a person with dementia, and protecting their self-esteem. Caregivers have found it helpful to use prompts and provide clear instructions, without removing the opportunity for the person to attempt activities. Team working is considered essential, and continuity of care is helpful where possible.

Strategies used by the respondents are summarized in Table 1 and described in the full report.

**Table 1:** Summary of strategies used.

Area of concern	Psychosocial approaches	Practical strategies	Professional processes	Team working
Lack of awareness in general	Person-centered Kindness Patience Perseverance Enabling Avoid confrontation	Prompts Clear instructions Watchfulness Time out	Continuity of care Flagging future issues Psychotherapy around denial Acknowledging the difficulties/care challenges Capacity assessments	Multidisciplinary team: healthcare, social care and family Community resources Safe spaces
Delays and disagreements	Reframe the problem: Offer to help ‘as I am younger’. Doing things differently, for another reason. Validate feelings Humor Use acceptable wording Emphasize potential benefits of confirming diagnosis Discussion about maintaining current quality of life	Act on concerns early Be informed Prompting Arrange ‘well person’ check Offer low level support initially Introduce care staff in different roles (sometimes without uniform)	Joint assessment with trusted professional Encourage diagnostic process anyway Pre-diagnostic counseling Allow time to build up trust Find the best person for difficult discussions Safeguarding referrals if indicated Provide support for family when difficult decisions faced Remote communication with family if needed	Peer support: engaging with other people with diagnosis Report poor care Education for family members Training for professionals
Safety	Turn the problem round: can you help me with this Distraction	Not going out alone Holding hands crossing road and giving narrative Careful planning train journeys Remove access to vehicle Arrange car journeys for enjoyment Cook together Technology in the home	Risk assessment Contingency planning Safeguarding alerts	Outside agencies for home safety assessments

#### 3.3.1 Managing delays and disagreements

Caregivers described continual encouragement (to attend a doctor’s appointment), to remind and persuade the person to accept support.

*“We suggested she had a well person check with the GP. I spoke to the GP prior to the appointment to share my concerns.”*

Clinicians described gently supporting the person to engage with assessment, giving the person time to come round to the idea that they need help, and using careful communication to find wording that is acceptable to that person. Homecare professionals described how care can be introduced gently and gradually.

*“Offering a low level of service i.e. bit of shopping, bit of cleaning, bit of general help to start off with to develop a trusting relationship over time.”*

To avoid disagreements, caregivers avoid confrontation where possible.

*“Never argue, it’s pointless.”*

For clinicians, working with the individual to build up trust is key. This needs to be closely tailored to the individual.

*“I think they know they have dementia but as I am not saying it, they are accepting and trusting that I am trying to get them help.”*

*“Frank and honest discussions with the person lacking insight can be surprisingly helpful but this takes experience to know when, how and who with. This can also easily antagonize.”*

Validation of the individual’s feelings is essential, and caregivers often use humor to lighten a situation. Peer support can be valuable.

*“Meeting other people with dementia who speak openly about their struggles and ways to address difficulties can help.”*

Clinicians and home care professionals working with the family offer support and practical advice, whilst also acknowledging the reality of how challenging this can be.

#### 3.3.2 Managing safety and safeguarding concerns

At home, doing things differently or doing things together are common solutions, with an emphasis on the benefits, such as no longer having to drive. Distraction can be useful to move away from a potentially dangerous situation. Changes in the home can include technology to improve safety, with the use of a video doorbell linked to the caregiver’s phone, or CCTV or sensor systems around the house.

*“Try to allow some independence but make the environment safe.”*

Clinicians make use of protocols and surveillance to address safety risks. These include multi-disciplinary team working with risk assessments and planning for contingencies *“…to support the family and ensure this support doesn’t break down”*.

### 3.4 What would help?

Caregivers would like help around earlier detection of symptoms and prompting by professionals to enable earlier diagnosis. Clinicians and homecare professionals suggested wider public information campaigns could highlight and address the delays in accessing support. Caregivers needed more support and closer working between families and professionals. Clinicians agreed that counseling resources for people with dementia and families could match that offered, for example, to cancer patients. More funding would provide better financial support for informal caregivers and allow professional homecare to start earlier to avoid crises. Education about awareness issues would be a helpful addition to clinician and homecare professional training programs. Dementia services may need to adapt in recognition of the barriers to obtaining support for people with dementia symptoms who lack insight into needs and who resist formal assessment and diagnosis.

### 3.5 Views from people with dementia

From the MIDAS interview transcripts, the people showing ‘extensive awareness’ noticeably had a good understanding of their diagnosis and were able to describe strategies that they used to manage everyday tasks such as writing notes, making lists, keeping a daily routine, rituals for remembering medication, using microwave meals instead of cooking, or stopping driving.

*“I said, ‘I’ve decided I’m going to stop driving. I think I’m going to be a danger on the road’.”*

Very few strategies were described by the people with ‘no evidence of awareness’.

*“I sit quiet and … tell myself, ‘Now there’s something I’ve got to do about …’.”*

The latter group also showed less concern about the future, and no particular worries were described.

*“Any worries? No, I don’t think so, no. I can’t think of anything.”*

*“I don’t think much about the future to be truthful.”*

In contrast, the more aware people discussed the future and the likelihood of needing residential care at some stage, with associated worries and sadness for some.

*“If she dies first then I’ve got things that I have to do and with a deteriorating…mental capacity that’s rather frightening.”*

*“I see the future as being pretty hopeless really.”*

Among those with better awareness, some people reflected on being aware of changes and reactions to being diagnosed with dementia.

*“It’s hard to sort of explain because it’s part of you wants to know and the other part doesn’t. Then you’re frightened of it…that erm you don’t know what erm cos I’ve never really known anybody with it.”*

*“To be honest I was devastated when I actually got the news. But I was also relieved. Because at first, I felt I wasn’t believed.”*

One individual with dementia gave this advice for other people who realize their memory is changing: “…*you should straight away go and see about it.”*

## 4 Discussion

The stakeholder consultation found that lack of awareness in people with dementia is a specific and significant problem recognized by informal caregivers, clinicians and homecare professionals. Whilst people who have good awareness are able to reflect on their condition, make plans and adopt strategies to manage ongoing activities, it is difficult to approach these issues with people who seem unaware of changes or deny that any help is needed. Lack of awareness can have serious consequences for people with dementia and their family members, as well as having an impact on professional workers.

Whilst there are known stresses inherent in dementia caregiving (Quinn et al., 2024), there can be additional challenges when caring for someone who lacks awareness (Ceolin et al., 2025). For homecare professionals and clinicians, best practice in person-centered care becomes difficult to achieve (Bailey et al., 2019). The existing care pathways (National Institute for Health and Care Excellence, 2018) do not adequately recognize the problems, and there can be obstacles to accessing appropriate care and support for this group of people with dementia. This can be further complicated if the main caregiver also lacks awareness or understanding of dementia-related changes in the individual they live with or care for (Boise et al., 1999).

Common strategies are based on a flexible and patient approach, allowing the person to build up trust with the care provider. A team approach is needed, exemplified by the ‘triangle of care’ (Hannan et al., 2016), with the family supported by and working with the clinical and social care teams, and with access to community resources. Clinicians often have central roles and responsibilities in making decisions at key points or crises and are reliant on good communication within the care network of families and homecare providers.

This can be very challenging. Caregivers described the stress, leading to exhaustion and the need for some time away. Lack of awareness of changes in social behavior can inadvertently lead to situations causing offence or embarrassment. This can create reluctance by the caregiver to initiate future social activities. Clinicians reported being blamed for difficult conversations and having their professional judgement questioned. Lack of awareness can be associated with neuropsychiatric symptoms (Tondelli et al., 2021) and homecare professionals described experiencing hostile environments when providing care, which can have implications for workforce sustainability. For people with dementia, awareness of the implications of a dementia diagnosis can bring sadness (Azocar et al., 2021), but lack of awareness can increase confusion and isolation, as well as numerous safety risks described by respondents. Optimal management may mean that various strategies need to be trialed before finding the best approach for a specific person and circumstance. An individual deemed to have capacity has the right to refuse a medical referral and reject care provision (Mental capacity act, 2005) and currently there is no straightforward solution for enabling people to get the right sort of support without a clinical assessment and diagnosis (National Institute for Health and Care Excellence, 2018). Similarly, an individual has the right to make decisions that might be considered unwise by others, assuming they have mental capacity. Sometimes informal caregivers and/or professionals are uncomfortable with a level of risk that is acceptable to the person with dementia, and which enables them to maintain a degree of independence and participation. The caregiver may also lack full awareness of the individual’s competence at times, leading to over-restrictive caring. This area is ethically complex (Marsh and Kelly, 2018). An individual should where possible be “allowed the dignity afforded by risk-taking” (Ibrahim and Davis, 2013, p. 189), but this can be difficult to balance with potential safety risks posed by lack of awareness (Starkstein et al., 2007), requiring close attention and tailoring to individual circumstances.

More information would be helpful for the general public, available to people with undiagnosed dementia and their family members around the time of emerging symptoms and signs of dementia, with signposting for advice for concerned relatives. Specific educational resources for informal caregivers and training for clinicians and homecare professionals would improve understanding of awareness issues and promote the sharing of effective strategies. Access to technological solutions could improve home safety for people who lack awareness and live alone. For example, personal budgets for dementia care could be used to fund equipment to help informal caregivers monitor safety in the home. A proactive approach could facilitate earlier identification of dementia and recognition of awareness difficulties in individuals. This approach could address better continuity of care and closer monitoring of vulnerable individuals. Documenting awareness status in primary and secondary care records using suitable clinical tools could help guide tailored care plans as well as benefit future research.

### 4.1 Areas for further research

Sometimes the caregiver has their own health problems and may lack capability and awareness for the caring role; this area could be investigated more fully. In a care home population of people with more severe dementia, changes in sensory awareness can become increasingly apparent (O’Shaughnessy et al., 2021). This can affect personal care needs such as continence management, requiring sensitive care to maintain the individual’s personal dignity and well-being, and could be discussed further in staff training programs. It can be difficult to distinguish whether apparent lack of awareness has a neurological or psychological basis (Prigatano et al., 2024) and unraveling this could help provide the relevant type of individual support.

### 4.2 Limitations of this consultation

The consultation involved a small sample of people from known networks, who may have been motivated to respond because they had experienced significant issues around lack of awareness. The collation of findings and selection of quotes was undertaken by a single researcher (the first author). Whilst involvement of a second researcher may have increased reliability it was not feasible given the resource limitations. Responses represent subjective experiences, and the survey was not designed to measure prevalence of adverse events, for example regarding safeguarding events related to lack of awareness, and there was little representation from social services. However, the draft report was shared with representatives from the consultation groups, to check the validity of the conclusions. Although survey respondents came from a wide regional area, ethnic diversity within the sample was limited, with respondents mainly from white ethnic groups.

## 5 Conclusion

Lack of awareness is a recognized problem in dementia care and can have serious consequences for the person with dementia and significant implications for effective care provision, yet there is little published research about outcomes or interventions. Further education about awareness would be helpful for informal and professional caregivers, and clinicians. Awareness status, adaptation of care pathways and appropriate care strategies should be explored further to improve support for people with dementia and care providers.

## Data Availability

The raw data supporting the conclusions of this article will be made available by the authors, without undue reservation. The full report of the stakeholder consultation is available at: Alexander (2025).

## Author disclaimer

The views expressed are those of the authors and not necessarily those of the Alzheimer’s Society, NIHR, or the Department of Health and Social Care. For the purpose of open access, the authors have applied a ‘Creative Commons Attribution (CC BY) license to any Author Accepted Manuscript version arising.

## Supplementary material

Data sheet 1 Survey questions.
