# Supplementary material for: Care challenges related to lack of awareness in people with dementia
Source: Front Dement. 2026 Feb 12;5:1770977. doi: 10.3389/frdem.2026.1770977 (PMC12936000; doi:10.3389/frdem.2026.1770977)
Supplement: Data sheet 1 — Survey questions. [file Data_Sheet_1.PDF]

frontiers

Supplementary Material

Survey questions

Informal caregivers

Sometimes people with dementia can seem unaware of changes due to dementia. This can be due to the disease, or as part of coping or denial about the condition. It might mean that they don't recognise memory problems, or over-estimate their ability to carry out everyday tasks.

1. Have you come across this in situations with the person you care for?

Not at all

Rarely

Sometimes

Often

Extremely often

2. Has a lack of awareness led to any delays in getting referral for diagnosis?

Not applicable, has seemed fully aware

No, lack of awareness did not contribute to any delays in referral

Not sure

Yes, caused some delay in referral (up to one year)

Yes, caused significant delay in referral (one year or more)

3. In your opinion, is the person you care for currently aware of having a diagnosis of dementia?

Yes, is definitely aware

Yes, probably

Not sure

No, I don't think so

No, seems completely unaware of the diagnosis

4. Has a lack of awareness resulted in any disagreements at home?

Not at all

Rarely

Sometimes

Often

Extremely often

5. Has a lack of awareness resulted in safety concerns of any kind at home?

Not at all

Rarely

Sometimes

Often

Extremely often

6. If you have noticed any difficulties due to lack of awareness, which areas have you been concerned about for the person you care for ? (Please tick as many as applicable)

Concerns about managing own medication

Concerns about cooking on own

Concerns about self-managing personal care

Concerns about safety of driving

Concerns about managing finances

Concerns about scams or rogue traders

Concerns about going out alone e.g. to the shops

None of these

7. Has a lack of awareness affected any of the following issues for the person with dementia? (Please tick as many as applicable)

Willingness to attend medical reviews

Willingness to engage with dementia support in the community

Managing usual social activities

Emotional response to others e.g. family members, friends

None of these

8. In your own words, what is your main concern, if any, about problems arising from lack of awareness in the person you care for?

9. How have you handled these situations? What has been the worst consequence?

10. What strategies have you found helpful? Do you have any other ideas or suggestions about the best way to manage these situations? What else would you need to help you manage better?

Clinicians

Sometimes people with dementia can seem unaware or lack insight about changes due to dementia. This can be due to the disease, or as part of coping or denial about the condition. It might mean that they don't recognise the diagnosis or memory problems, or they over-estimate their ability to carry out everyday tasks and decline appropriate support.

1. Have you come across this in situations with any person with dementia you have cared for?

Not at all

Rarely

Sometimes

Often

Extremely often

2. Has lack of awareness in an individual led to any delays in diagnosing dementia?

No, not to my knowledge

Yes, but rarely

Yes, in some cases

Yes, this often happens

Yes, this is extremely common

3. Has lack of awareness led to an individual having difficulties in accepting the diagnosis of dementia?

Not at all

Rarely

Sometimes

Often

Extremely often

4. Has lack of awareness in an individual led to difficulties in discussing dementia care and/or prescribing appropriate medication?

Not at all

Rarely

Sometimes

Often

Extremely often

5. Has a lack of awareness in an individual with dementia led to any difficulties in other areas of health management such as managing continence, nutrition, treating long term conditions such as diabetes, or recognising non-dementia symptoms?

No, not to my knowledge

Yes, but rarely

Yes, in some cases

Yes, this often happens

Yes, this is extremely common

6. Has a lack of awareness in an individual with dementia resulted in any significant safety or safeguarding issues?

No, not to my knowledge

Yes, but rarely

Yes, in some cases

Yes, this often happens

Yes, this is extremely common

7. Do you think that lack of awareness has contributed to breakdown of home care (paid or unpaid) for any of the people you care for?

No, not to my knowledge

Yes, but rarely

Yes, in some cases

Yes, this often happens

Yes, this is extremely common

8. In your own words, what are the main problems you have encountered, if any, due to low awareness in a person with dementia you have cared for?

9. How have you handled these situations? What has been the worst consequence?

10. What strategies have you found helpful? Do you have any other ideas or suggestions about the best way to manage these situations? What else would you need to help you manage better?

Homecare and social care professionals

Sometimes people with dementia can seem unaware of changes due to dementia. This can be due to the disease, or as part of coping or denial about the condition. It might mean that they don't recognise the diagnosis or memory problems, or they over-estimate their ability to carry out everyday tasks and decline appropriate support.

1. Have you come across this in situations with any person with dementia you have cared for?

Not at all

Rarely

Sometimes

Often

Extremely often

2. Has lack of awareness in an individual with dementia led to any delays in initiating home care?

Not at all

Rarely

Sometimes

Often

Extremely often

3. Has lack of awareness in an individual with dementia restricted the type of care you can provide?

Not at all

Rarely

Sometimes

Often

Extremely often

4. Has lack of awareness in an individual with dementia resulted in disagreements or conflict about the help needed during home care?

Not at all

Rarely

Sometimes

Often

Extremely often

5. If you have noticed a lack of awareness, which areas of care provision have been problematic? (Please tick as many as applicable)

Gaining access to home

Assisting with personal care

Supervising medication

Assisting with meals

Discussing equipment needs

Discussing future plans e.g. Lasting Power of Attorney, living situation

Discussing social activities and community support

None of these

6. Has a lack of awareness in an individual with dementia resulted in any significant safety or safeguarding issues?

Not at all

Rarely

Sometimes

Often

Extremely often

7. Do you think that lack of awareness has contributed to breakdown of home care for any of your clients?

No, definitely not

No, was not the main reason

Not sure

Yes, was a factor in care breakdown

Yes, definitely

8. In your own words, what are the main problems you have encountered, if any, due to low awareness in a client with dementia?

9. How have you handled these situations? What has been the worst consequence?

10. What strategies have you found helpful? Do you have any other ideas or suggestions about the best way to manage these situations? What else would you need to help you manage better?
